# SHAPED – Swiss Health Alliance for Interprofessional Education: A project report on the student-led development and implementation of intra- and extracurricular interprofessional learning activities

**DOI:** 10.3205/zma001787

**Published:** 2025-11-17

**Authors:** Lucas Büsser, Corina Zweifel, Jana Bühler, Felice Hess, Carmen Zürcher, Fanny Mulder

**Affiliations:** 1 Swiss Health Alliance for Interprofessional Education, Bern, Switzerland; 2 University of Basel, Faculty of Medicine, Basel, Switzerland; 3 University of Applied Sciences Bern (BFH), School of Health Professions, Bern, Switzerland; 4 XUND Bildungszentrum Gesundheit Zentralschweiz, Luzern, Switzerland; 5 University of Bern, Institute of Primary Health Care (BIHAM), Bern, Switzerland

**Keywords:** interprofessional education, health professions education, medical education, student leadership, interprofessional collaboration

## Abstract

**Description of project/objectives:**

Internationally, healthcare students call for more interprofessional education (IPE) and in Switzerland some of them formed the *Swiss Health Alliance for Interprofessional Education* (SHAPED, [https://www.shaped-ip.ch/]). As a students’ and young professionals’ association, SHAPED has developed multiple participatory, realistic, and enjoyable interprofessional (IP) learning activities. This project report describes the development as well as the implementation of these IP learning activities, initially in the extra-, and more recently also in the intracurricular setting. Additionally, it evaluates their benefits to students.

**Results:**

From 2020 to 2024, more than 2,000 students stemming from ten different healthcare professions participated in IP learning activities developed by SHAPED. Quantitative analysis using the *Students Perception of Interprofessional Clinical Education Revised* (SPICE-R) instrument showed a significant increase in students’ perception of IPE pre-to-post participation, with a medium effect size (*t*(540)=-13.4, *p*<.001, *d*=0.574). This increase in perception was similar for both, the intra- and extracurricular setting.

Qualitative analysis confirmed that while refinement of format and content was sometimes indicated, most of the participants appreciated the interactive nature of SHAPED’s activities and enjoyed learning new aspects about the roles and responsibilities of other healthcare professions.

**Conclusion:**

SHAPED is an exemplary project showcasing how the student-led development and implementation of IP learning activities can overcome many barriers faced by IPE-inclined faculty and why it should thus be considered as a valid alternative to advance IPE everywhere.

## Zusammenfassung

**Beschreibung des Projekts/Ziele:**

International rufen Gesundheitsstudierende zu mehr interprofessioneller Ausbildung (*engl. Interprofessional Education*, IPE) auf und in der Schweiz haben sich einige davon als *Swiss Health Alliance for Interprofessional Education* (SHAPED, [https://www.shaped-ip.ch/]) zusammengeschlossen. Als Verein von Studierenden und Berufseinsteigenden hat SHAPED mehrere erlebbare, realitätsnahe und freude-bereitende interprofessionelle (IP) Lernaktivitäten entwickelt. Dieser Projektbericht beschreibt die Entwicklung und Umsetzung dieser IP-Lernaktivitäten, initial im extra- und nun auch im intracurricularen Umfeld. Zusätzlich werden die Vorteile solcher Aktivitäten für Studierende evaluiert.

**Resultate:**

Von 2020 bis 2024 haben mehr als 2.000 Studierende aus zehn unterschiedlichen Gesundheitsberufen an IP Lernaktivtäten von SHAPED teilgenommen. Die quantitative Analyse mittels des *Students Perception of Interprofessional Clinical Education Revised* (SPICE-R) Instruments zeigte eine signifikante Verbesserung der studentischen Wahrnehmung von IPE durch die Teilnahme an den von SHAPED entwickelten IP-Lernaktivitäten mit einer mittleren Effektgrösse (*t*(540)=-13.4, *p*<.001, *d*=0.574). Diese Verbesserung in der Wahrnehmung war ähnlich im intra- wie extracurricularen Kontext. Eine qualitative Analyse bestätigte, dass zwar manchmal eine Verfeinerung des Formats und der Inhalte angebracht war, die meisten Teilnehmenden jedoch den interaktiven Charakter der IP-Lernaktivitäten schätzten und es genossen, neue Aspekte über die Rollen und Verantwortlichkeiten anderer Gesundheitsberufe zu lernen.

**Schlussfolgerung:**

SHAPED zeigt exemplarisch, wie die von Studierenden geleitete Entwicklung und Umsetzung von IP-Lernaktivitäten viele Hindernisse überwinden kann, mit denen IPE-orientierte Lehrkräfte konfrontiert sind. Aus diesem Grund sollten solche von Studierenden entwickelte und umgesetzte IP-Lernaktivitäten als gewinnbringende Alternativen angesehen werden, um IPE überall voranzubringen.

## 1. Introduction

### 1.1. The importance of interprofessional education

Interprofessional collaboration (IPC; alternatively collaborative practice) is considered a key element to successfully meet the healthcare challenges of the 21^st^ century [1]. IPC does not only provide better patient care, but is becoming even more important in times of workforce shortages [2] and rising healthcare costs [3]. To achieve functioning IPC, interprofessional education (IPE) is crucial [3]. According to the World Health Organization, IPE *“occurs when students from two or more professions learn about, from and with each other to enable effective collaboration and improve health outcomes”* [3].

### 1.2. Description of needs

Behrend et al. [4] reported that medical students rate the relevance of IPC as part of their education much higher than its actual implementation in the curriculum, showcasing the need for more IPE from a student perspective.

Reports of gaps in IPE opportunities for healthcare professionals in Switzerland and beyond [5], [6], [7] further demonstrate that this need is not always met. During the COVID-19 pandemic, this situation was worsened by the cancellation of various programs and the transition to online and/or blended learning taking place globally [8], [9]. At a time when IPC became more important than ever [10], the unavoidable restructuring of the curricula of thousands of healthcare students disrupted established IPE programs.

### 1.3. Barriers and enablers to IPE

Already a decade ago, Lawlis and colleagues identified a multitude of barriers and enablers that influence sustainable IPE in higher education institutions [11]. The ongoing lack of pre-graduate IPE in Europe – particularly in terms of involving all healthcare professions and starting early on in the curriculum [6] – indicates that the barriers continue to prevail. A recent study published in this journal underscored some of them, such as a lack of interfaculty collaboration, as contributors to the limited availability of IPE offerings [12]. Table 1 (Tab. 1) shows how Lawlis et al. categorized these barriers on three levels: government and professional, institution, and individual. 

### 1.4. State of IPE in Switzerland and best practices in IPE

Over recent years, several IPE initiatives have been implemented in Switzerland [12], [13], [14]. Nonetheless, gaps remain, leading to calls for increased institutionalization of IPE projects [5], [12]. 

It is known that participation of healthcare students in programs that enable them to interact in a safe IP learning environment can increase their knowledge and skills, among other, regarding communication, teamwork, and role understanding [15], [16]. Together with values & ethics, these constitute the four core competencies for IPC as outlined by the Interprofessional Education Collaborative [17].

In their recent realist review, Maddock et al. [16] identified six design and three learning features that enhance IPE interventions, including *“each profession could offer knowledge and skills the other profession could not, to solve the problem”* and *“a clinically challenging case, pertinent to all involved professions”*. Furthermore, best practices guides in IPE exist, such as the one by Willgerodt et al. [18], which, among recommendations, calls for collaboration with existing organizations, and the establishment of an interprofessional (IP) team to ensure relevancy and authenticity.

### 1.5. Student leadership in IPE

Also in other parts of the world students recognized the lack of IP learning opportunities and consequently formed student-led initiatives [19]. Already in 2008 Hoffman et al. recognized the advantages of student leadership in IPE [20]: Students not only provide a resource-friendly alternative, but they have firsthand knowledge of the needs of their peers. Furthermore, near-peer-teaching has been shown to foster a safe learning atmosphere and meet learners on their cognitive levels [21] – benefits that student-led IPE programs can draw upon.

### 1.6. Extracurricular teaching and learning activities

In their recent integrative literature review, Kim et al. outlined multiple learning outcomes of extracurricular activities for medical students and found that participation in extracurricular activities increased academic performance, deepened understanding and interest in specific specialties, and helped professional development [22]. Outside of medicine, extracurricular activities were found to increase nursing skills [23] and enhance pharmacy students’ ability to apply clinical knowledge to case-based scenarios [24]. Finally, viewed from an IPE perspective, a previously described project named interTUT [25] identified its *extracurricular* nature as a key facilitator to implementation, helping it circumvent some of the barriers mentioned above.

## 2. Project description

### 2.1. Aim of the project

To address the inadequate IPE offerings and overcome barriers to IPE, students and young professionals from different fields of healthcare founded the *Swiss Health Alliance for Interprofessional Education *(SHAPED, [https://www.shaped-ip.ch/]) in 2020. SHAPED aims to strengthen IPE and IPC in the Swiss healthcare system. By uniting like-minded students and young professionals from different professional backgrounds and across institutions, many of the aforementioned barriers, especially on an individual level, can be overcome (marked bold in table 1 (Tab. 1)). Furthermore, with its organizational structure as a voluntary-based association, SHAPED effectively utilizes many of the outlined enablers on a government and professional, as well as an individual level (also marked bold in table 1 (Tab. 1)). As an example, by relying on voluntary work, the enthusiasm of SHAPED facilitators is a prerequisite. Furthermore, by working hand in hand to build a new association, a shared interprofessional vision is guaranteed. Emphasizing on working in interprofessional teams within the association itself, shared ownership, and seeing the value of and showing respect towards other healthcare profession(al)s are integral to working within SHAPED. However, on all levels barriers such as lack/limited financial resources, lack/limited institutional support, and high workload for facilitators remain.

This project report aims to evaluate whether a student- and young professional-led association (i.e. SHAPED) can develop, implement and sustain IPE activities that benefit students.

- Hypothesis 1: SHAPED can develop and conduct extracurricular IPE activities.
- Hypothesis 2: SHAPED can develop and conduct intracurricular IPE activities.
- Hypothesis 3: The IPE activities of SHAPED provide measurable benefits to participants.

### 2.2. Methodical approach

**Figure 1:**
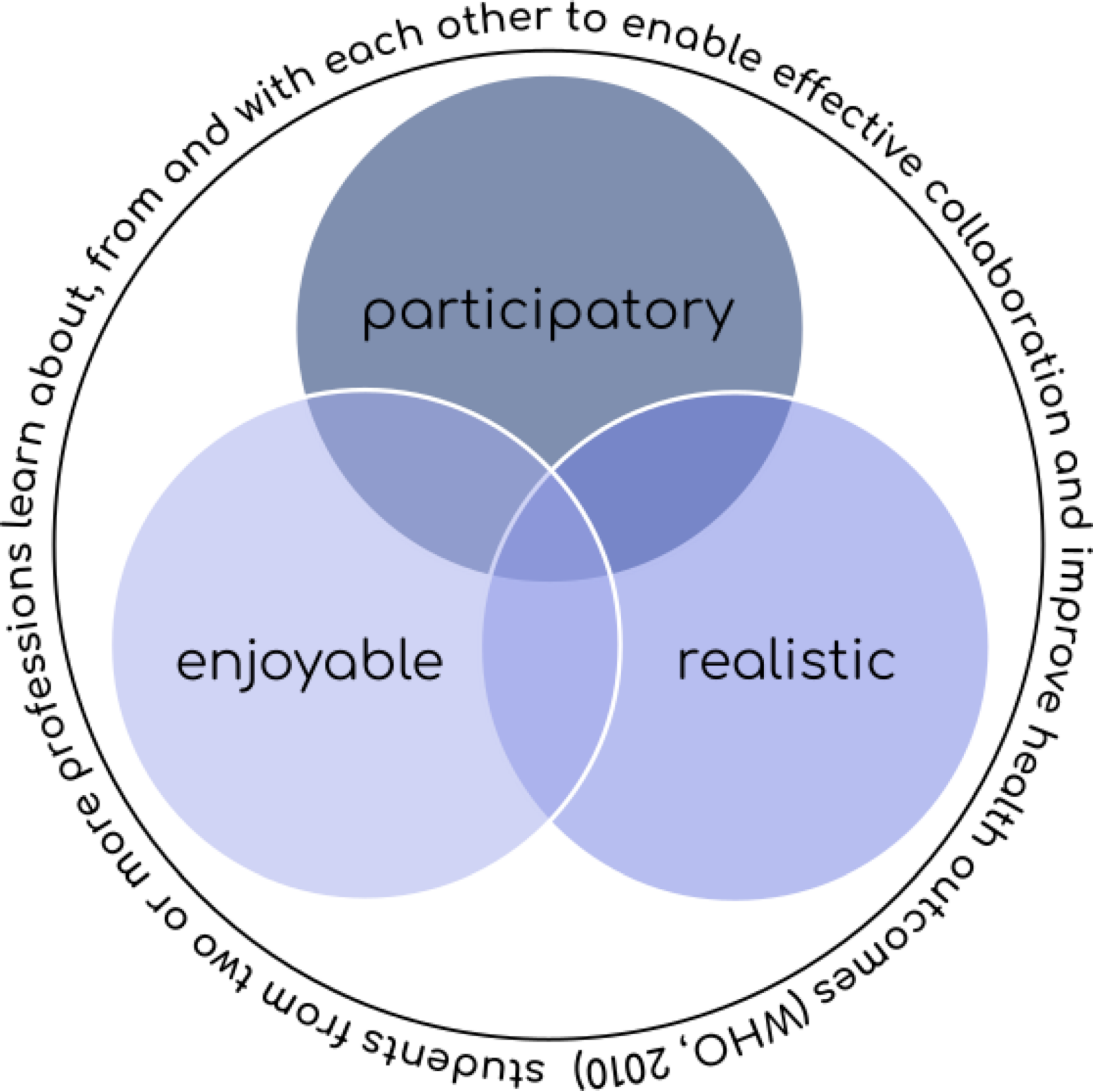
The three quality attributes guiding SHAPED’s development of interprofessional learning activities

SHAPED develops its IP learning activities based on the three quality attributes displayed in figure 1 (Fig. 1) and by following the aforementioned best practices for IPE [16], [18]. The quality attributes were derived from a position paper on IPE based in literature and student-expert opinions of the collaborating seven Swiss healthcare students’ associations [26]. This position paper emphasizes that from a student perspective, “good” IPE activities should be *participatory* (i.e. students should actively participate in the activity) and *realistic* (i.e. activities should reflect real life scenarios, if possible). These conditions are in line with *situated* and *experiential learning* often described in IPE literature [27] and match recommendations by Maddock et al. [16]. Furthermore, there is evidence that fun and enjoyment benefit adult learning [28], resulting in* enjoyable* (i.e. having fun during IP courses and thus enhancing engagement and motivation to learn) becoming the third quality attribute to guide the development of SHAPED’s IPE activities.

#### 2.2.1. Development of extracurricular IP learning activities

Extracurricular IPE activities were the initial focus of SHAPED, due to their higher perceived feasibility of implementation [25], especially during the pandemic. In IP project groups – thereby following recommendations in literature to ensure the relevance and authenticity of IP activities [18] – SHAPED members discussed and conceptualized different project ideas, guided by the quality attributes outlined in figure 1 (Fig. 1). 

The *Interprofessional Case Discussions* (ICDs) were the first IP learning activity developed by SHAPED. 

ICDs are held in small groups of 8-10 students from different healthcare professions that come together to solve a patient case in a “murder mystery”-format. Participants receive profession-specific scripts prepared by SHAPED members, which contain only partial information about the case, mirroring the way information is typically provided in clinical practice. Thus, effective communication and IP teamwork are required to find the murderer (i.e. the disease) and start the correct treatment plan in time. Since the scripts contain the necessary background information, more advanced students can also take on the role of a different profession (e.g. a medical student working with the physiotherapy script) and thus learn even more about other professions’ roles and responsibilities. The ICDs are largely based on the contact theory, first developed by Allport [29] and often used in IPE since [27], [30]. Key to supporting positive group interaction and thus IPC is not only bringing individuals from different backgrounds together, but ensuring certain conditions such as a common goal (i.e. solving the ICD), a cooperative atmosphere (i.e. no script alone contains all the information needed), institutional support (i.e. facilitation by a SHAPED-facilitator), and equal status (i.e. all participants receive a profession-specific script) are met [27], [29], [30]. 

The *shadowing* program was initiated as a second learning activity. Here students are invited to accompany a healthcare provider from another profession for a half or a full day. The conceptual framework of the shadowing lies in experiential learning theory, which was first described by Kolb [31] more than 40 years ago. It involves the cyclical 4-stage model of

1. concrete experience (i.e. following another healthcare professional for one day),
2. reflective observation (i.e. filling out the reflective learning material provided by SHAPED),
3. abstract conceptualization (i.e. writing a report on what has been learned), and
4. active experimentation (i.e. applying new ideas to daily practice in one’s own profession) [27], [32].

Following the initiative of SHAPED and the support of motivated tutors – practicing healthcare professionals found through a snowballing system and newsletters of professionals’ associations – students got to experience the daily work of another profession.

The *IP café* is SHAPED’s latest extracurricular activity and the first one aimed at young healthcare professionals (i.e. post-graduates). It was initiated in spring 2023 and is based on the interprofessional socialization theory described by Khalili et al. [33]. Post-graduates have already formed a professional identity during their often mono-professional education, but tend to lack the interprofessional identity needed for successful IPC [33]. As a result, young healthcare professionals experience many barriers to IPC, when entering the workforce. These include other healthcare professionals identifying more strongly with their own profession than with the IP team as a whole, undervaluing of the role of other professionals, and non-cooperative inter-group communication [34]. The* IP café* aims to increase interprofessional socialization by enabling cross-professional interactions, where pre-existing views are intentionally and critically challenged through discussion in a safe environment (i.e. *stage 1: breaking down barriers*). Later on, participants are encouraged to discuss personal experiences, allowing a shared understanding of each other’s roles, knowledge and skills to emerge. This ultimately leads participants to explore how to engage in effective IPC in clinical practice (i.e. *stage 2: IP role learning – IP collaboration*) [33]. 

#### 2.2.2. Development of intracurricular IP learning activities

Literature shows that the development of smaller scale IPE activities can be an effective mean of their integration into the curriculum [18]. The ICDs meet these criteria, but when introduced into established programs, their small-group nature encountered some of the barriers commonly associated with IPE, such as lack/limit of financial resources and a high workload for faculty [11]. To ease the burden on educational institutions and to overcome these barriers with the goal of reaching even more healthcare students, SHAPED started to adapt and tailor its IP learning activities accordingly. As a result, the *IP challenge* and the* IP pub quiz* were developed. The element of gamification, which has previously shown to improve collaboration (i.e. teamwork) of participants [35], was purposefully included in both activities. By providing clear and relevant goals, connecting participants to support each other, and providing immediate feedback and positive reinforcement, SHAPED followed many principles of gamification outlined by a recent systematic review [36]. The *IP challenge* was developed for early pre-graduate students and lets them discover each other’s roles, competencies, and responsibilities. Similar IPE interventions have shown to reduce prejudice in pre-graduates [37] and improve students’ attitudes towards other professions [38], [39]. The IP challenge takes place in classes of 40-50 students, that are first divided into monoprofessional groups and later reorganized into interprofessional teams. Students examine prejudices associated with various professions, discover the wide range of careers in healthcare, and explore with whom they will work together most closely in the future. The *IP pub quiz* targets more advanced students, and in addition to the goals of the IP challenge, focuses on IP teamwork and IP communication, two core IPC competency domains [17]. It takes place in a lecture hall with 100-150 students, seated in IP groups of 5-10 students. A SHAPED facilitator acts as the “quizmaster” and leads students through a patient case interspersed with various questions. The goal of each IP group is to answer the most questions correctly by combining their knowledge and through IP teamwork.

#### 2.2.3. Implementation of extracurricular IPE activities

In November 2020, SHAPED’s very first ICD took place as an extracurricular activity in an online setting. Following best-practice recommendations and through innovative use of technology, this format *“allow[ed] students who otherwise would not be able to participate to engage in IP activities”* [29], despite the pandemic. In spring 2021, the *shadowing* program started, sparking interest among healthcare professionals who readily agreed to become tutors and host students. As the latest extracurricular activity, and the first aimed at post-graduate students, the* IP café* was launched in Bern in spring 2023. 

SHAPED’s extracurricular IPE activities were promoted through its newsletter, its website, and social media (namely Instagram, *@shaped_ip*). Additionally, collaborations with student associations and educational institutions were sought to advertise the events, as recommended [18]. Furthermore, partnerships with local professionals’ associations ensured the necessary funding for events such as the *IP café*.

#### 2.2.4. Implementation of intracurricular IPE activities

Due to the promotion of SHAPED’s extracurricular activities at universities, faculty members became aware of the association. By engaging with key stakeholders such as IP program leaders and actively seeking organizational collaboration – thereby following best-practice advice [18] – integration of SHAPED’s IPE activities into the curriculum of healthcare students was achieved. Since January 2022, SHAPED regularly facilitates ICDs, the *IP challenge*, and the *IP pub quiz* at four educational institutions in Switzerland.

### 2.3. Evaluation of SHAPED’s IPE activities 

Since the beginning SHAPED has deployed its own Research & Assessment team to evaluate and assure the quality of its work, and to continuously improve its activities. During the development phase of the different IPE activities, open-ended question feedback form participants took centre stage, as this has been reported as a critical resource for reflecting on and improving teaching [40]. For this report, participants comments on what they liked and what could be improved with regards to SHAPED’s IPE activities were analysed with MAXQDA^®^ software (version 24.2.0), using a step-by-step process outlined by Naeem et al. [41]. An inductive approach was employed to develop individual keywords, codes and themes. Since grounded theory was not used, data saturation was not required.

After successful implementation, a quantitative approach to the evaluation of the IPE activities was established, using a pre-post design. Unfortunately, validated questionnaires for the evaluation of IPE activities in German-speaking countries are lacking [42]. Thus, considering feasibility and applicability in a multilingual country such as Switzerland, the validated Student Perception of Interprofessional Clinical Education Revised (SPICE-R) self-report instrument was used in its original English version [43]. It was chosen due to its brevity, which is linked to a higher response rate [44], as well as its advantage over other tools [43]. Students participating in the ICDs, the *IP pub quiz*, the *IP challenge* and the *shadowing* were provided with a link/QR-code leading to an anonymized online pre- and post-questionnaire. Participation was voluntary, and for intracurricular IP learning activities, it had no effect on the academic progress of students. Quantitative data was analysed using Jamovi^®^ software (version 2.3.28). Overall pre- to post-comparison was conducted using a paired t-test. For comparison between the extra- and intracurricular setting a two-way repeated measured analysis of variance (ANOVA) was applied, due to its results being readily understood and easily communicated [45].

## 3. Results

### 3.1. Participation in intra- and extracurricular IPE activities

Since its founding in fall of 2020 until December 2024 more than 2,000 healthcare students and young professionals participated in one of SHAPED’s IP learning activities. Table 2 (Tab. 2) lists the number of participants in either the extra- or intracurricular setting per year. While SHAPED initially focused on extracurricular activities, the number of participating students increased significantly after their incorporation into the curriculum in 2022.

### 3.2. Benefits to participants – SPICE-R

#### 3.2.1. Change in perception on IPE

From fall 2020 to February 2025 a total of 541 completed pre- and post- SPICE-R-questionnaires could be matched with certainty (estimated response rate at least 25%). They stemmed from participants of ten different healthcare professions with table 3 (Tab. 3) displaying their characteristics.

Taken together, SPICE-R scores of participants of all IP learning activities significantly improved from pre-(*M*=3.96; *SD*=0.38) to post-IP learning activity (*M*=4.17; *SD*=0.46; *t*(540)=-13.4, *p*<.001), with a medium effect size (*d*=0.574).

#### 3.2.2. Intra- vs. extracurricular IP learning activities 

**Figure 2:**
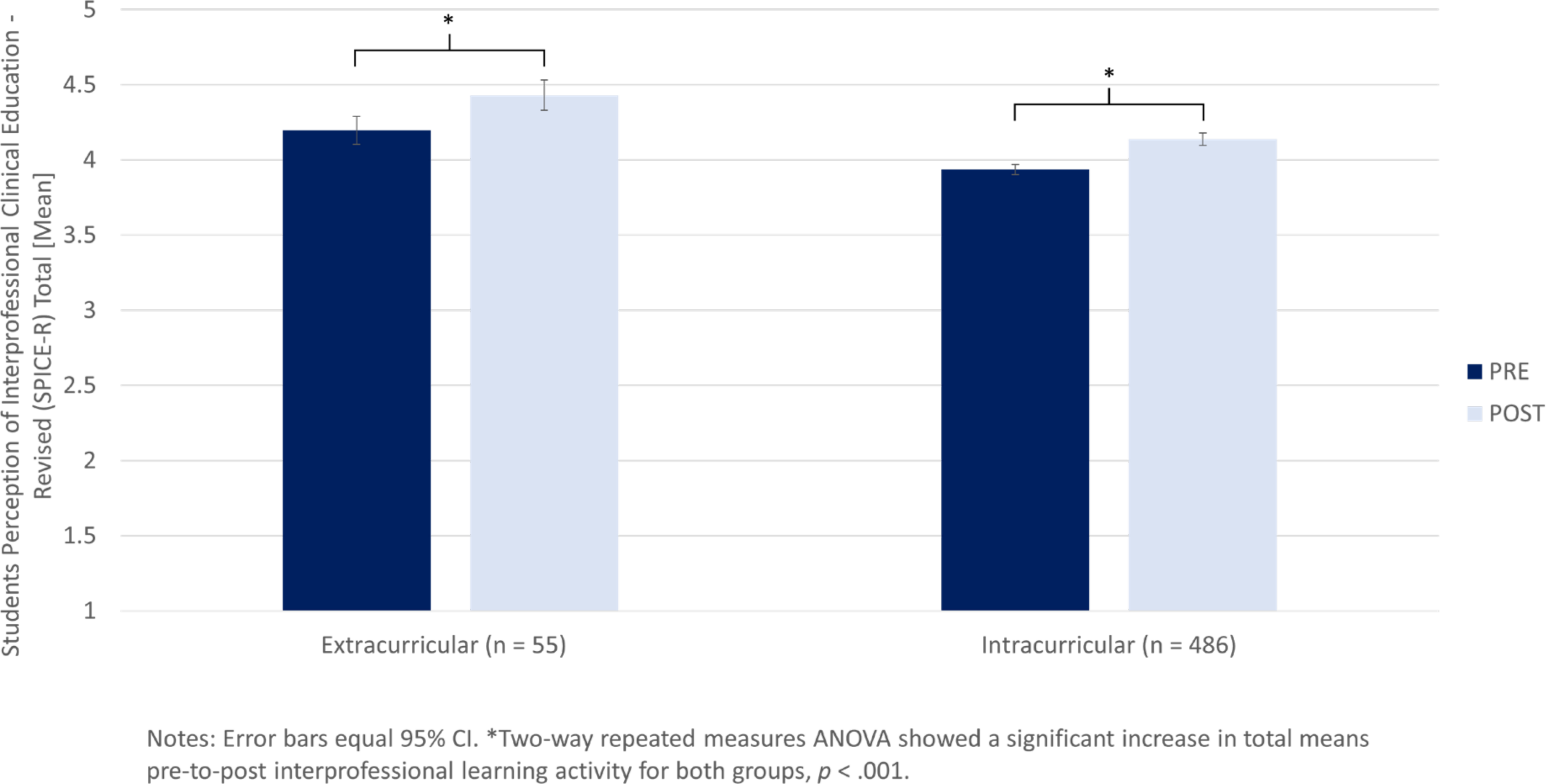
Change in participants’ perception on interprofessional education

As visualized in figure 2 (Fig. 2), a two way repeated measures ANOVA confirmed the positive main effect of SHAPED’s IP learning activities (pre-to-post) on the SPICE-R score (*F*(1,539)=73.527, *p*<.001). At the same time it did not show a significant interaction with the setting (*F*(1,539)=0.407, *p*=.524), suggesting that improvement for intra- and extracurricular activities was similar. However, overall scores were higher for participants in extra- compared to intracurricular activities (*F*(1,539)=27.5, *p*<.001). 

### 3.3. Benefits to participants|analysis of participants written feedback

From the analysis of the 161 answered qualitative feedback forms (response rate of approximately 35%), three themes emerged similarly for both, intra- and extracurricular activities:

1. *Content and format of activities *(positive aspects and suggestions for improvement),
2. *IPC and interactivity* (positive aspects and suggestions for improvement), and
3. *knowledge gain.*

#### 3.3.1. Content and format of activities (positive aspects and suggestions for improvement)

Participants found SHAPED’s IPE activities interesting and especially enjoyed the gamification aspect of the IP pub quiz. A lot of them would not change anything and found the overall environment encouraging. The patient cases (for the ICDs and IP pub quiz) were deemed quite realistic, and it was easy to follow with the structured scripts (ICDs). 

*“I liked the interactive method, following a clinical case and its evolution with questions” - participant of intracurricular IP pub quiz*

However, some participants mentioned that the IP pub quiz was too long, where others said that some part of the activity should be discussed more in-depth. For the ICDs, some participants highlighted that they would prefer a patient case that is more difficult, while others pointed out that some professions were not highlighted enough and could be more involved by expanding the cases. Finally, some participants suggested to take a deeper look into IP conflicts during patient care. 

*“Highlighting more the types of conflicts/challenges that may arise between different professions (as interests sometimes diverge…) [could further improve this activity]” - participant of extracurricular ICD*

#### 3.3.2. IPC and interactivity (positive aspects and suggestions for improvement)

Most participants mentioned that they liked the interaction during the activities, such as everyone being able to answer during the IP pub quiz. They also felt that they were building together as a team in order to help the patients and found the exchange with members of different healthcare professions extremely valuable. 

*“I liked the engaging discussion with the other professions from which one could learn a lot”*

*- participant of extracurricular ICD*

Some participants, however, perceived the groups as too big to allow fruitful IPC. Also, depending on the professional background of participants in the team, some questions were found inadequate and hard to answer. Furthermore, some participants would have liked to interact even more with members of their team and, for instance, not only discuss questions during the IP pub quiz. 

*“More interpersonal interactivity during the presentation [would be a way to further improve this activity], but it is understandable given the size of the audience” - participant of intracurricular IP pub quiz*

#### 3.3.3. Knowledge gain

A lot of participants emphasized that they learned something new about other participating professions during SHAPED’s activities. They enjoyed expanding their knowledge on the roles and responsibilities (incl. competencies and knowledge) of other healthcare professionals and different aspects of their daily life.

*“The best part was that my perspective was broadened. I gained insight into professional groups that I would never have considered or found relevant in this context.” - participant of extracurricular ICD*

## 4. Discussion

SHAPED managed to overcome multiple barriers that previously hindered IPE, by forming a students’ and young professionals’ association with no ties to one specific educational institution and/or region. With its members seeing each other as equals, regardless of their professional background, and by working in a truly interprofessional fashion, SHAPED manages to live an IP culture. As a result, the association has achieved the development of multiple IP learning activities and implemented them not only in the extra- but through collaborations with educational institutions also in the *intracurricular* context. Evaluation data shows that these activities, which adhere to quality attributes and best practices in IPE, yield a measurable benefit to participating students.

### 4.1. Extracurricular IPE activities

Hypothesis 1 is supported by the findings of this project report: a students’ and young professionals’ association such as SHAPED can develop and implement extracurricular IPE activities, such as online ICDs, the Shadowing program, and the* IP café* outlined above. This was also shown by other student-initiated projects [46], [47], but within Switzerland, no similar initiatives were previously reported. 

Starting with extracurricular IPE activities was likely an enabler, working in SHAPED’s favour. The extracurricular nature of other IPE projects has been noted as a key to success, as it *“bypasses many of the barriers faced by most undergraduate IPE programs”* [48]. For instance, the implementation of extracurricular IPE activities does not need major restructuring of profession-specific curricula [49]. 

Additionally, due to the reduced availability of other extracurricular activities during the COVID-19 pandemic [50], there was an opportunity for new initiatives like SHAPED to emerge and attract participants. This likely helped SHAPED get off the ground and transform its ideas into actions. Finally, a unifying effect of the pandemic on healthcare students was reported [51], which might be supported by the fact that SHAPED was founded during that time and participation in extracurricular activities was higher during the pandemic, than afterwards.

### 4.2. Intracurricular IPE activities

Likewise, Hypothesis 2 appears to be validated: a students’ and young professionals’ association such as SHAPED can develop and implement intracurricular IPE activities such as the in-person ICDs, the* IP challenge*, and the *IP pub quiz*. As outlined in chapter 2.2, by following best practices and due to the nature of SHAPED as an independent students’ and young professionals’ association many IPE-enablers could be tapped and barriers that hinder intracurricular IPE implementation were overcome (marked bold in table 1 (Tab. 1)). As previously reported [18], starting with the development of smaller scale IP learning activities (such as the ICDs) likely increased the chances of integration into the curricula. However, this can only be achieved with the help of partner institutions. By following lessons learned by others and engaging with key stakeholders [29], SHAPED managed after only two years to integrate its IPE activities into the curriculum of educational institutions, thereby significantly increasing the number of participating students. 

In pursuit of a more independent approach, and in contrast with other student-developed and -led IPE programs [52], [53], SHAPED did not originate from a university or institution and was not established in collaboration with faculty. Nonetheless, this project report demonstrates that it is possible to incorporate student-initiated IPE projects into the established curriculum of healthcare students.

### 4.3. Benefits to participants

Finally, this project report provides evidence that IPE activities developed and implemented by a students’ and young professionals’ association such as SHAPED can have a measurable benefit for participants, backing hypothesis 3.

Participants’ high base-line scores of SPICE-R indicate that their perception of IPE is positive, already before they partake in an IPE activity. This positive perception has been widely reported before [54], [55] and showcases that the current generation of healthcare students acknowledges the need for IP education and collaboration. However, through partaking in SHAPED’s IPE activities, these positive perceptions were further increased. This is in line with findings of a recent systematic review which demonstrated that *“IPE was effective in improving both pre-licensure learners and professionals’ attitudes toward other disciplines and the value placed on a team-based approach for improving patient outcomes”* [56]. 

Interestingly, while participants of extracurricular activities showed a generally more positive attitude towards IPE, there was no significant difference in benefit between the two groups. This counters the possible selection-bias, which could stem from extracurricular activities primarily attracting IP interested participants. Thus, demonstrating that also the average student population benefits from SHAPED’s activities. While previous evaluations of student-developed IPE activities showed benefits to students [53], a limiting factor was the lack of a validated assessment tool. By applying the SPICE-R instrument, this could be overcome in this project report.

The qualitative evaluation concurred in both settings that participants not only enjoyed SHAPED’s IPE activities, but found them beneficial, especially regarding their understanding of the roles and responsibilities of other healthcare professionals. The participatory aspect of the activities, the gamification in the *IP pub quiz*, as well as the positively perceived learning environment were frequently pointed out by participants. This is in line with the benefits of student leadership reported before [20], [21]. Feedback was also given to further improve the IPE activities, such as adapting patient cases, or further increase interactivity. Due to the independent nature of SHAPED and the flexibility of its project teams, much of the feedback could already be implemented, catering to the needs of the students immediately. 

Taken together evaluation data supports the use of the quality attributes outlined in figure 1 (Fig. 1) and following best-practice guides when developing new IPE activities.

### 4.4. Limitations of this project

The results of this project report need to be viewed considering the following limitations. The low estimated response rates for both the quantitative and qualitative evaluations raise the possibility of non-response bias. For the SPICE-R, this is partly due to the focus on paired data analysis, as individual response rates were higher for the pre- and post-questionnaires separately. Furthermore, while short-term effects of the IPE activities on participants’ perceptions of IPE could be demonstrated, mid- to long-term effects remain unclear, which is a frequent shortcoming of IPE research [56]. 

Regarding the qualitative evaluation, the type of questions deployed favoured short answers, leading to less constructive feedback. For future evaluations of IPE activities, more elaborate questions might lead to even deeper insights and additional themes emerging.

Additionally, the deployment of multiple independent project teams within SHAPED that developed the IPE activities led to the use of multiple conceptual frameworks, limiting generalizability of findings across the different IPE activities.

On a broader scale, while being an independent students’ and young professionals’ association has been an enabler of this project, the lack of direct affiliation with academic institutions limits the human and financial resources of SHAPED, as well as access to knowledge and skills regarding education and research. As a result, scalability of this project is limited. Furthermore, SHAPED’s members are students and young professionals putting their efforts into the project during their spare time, resulting in a high workload. This in turn requires an even higher intrinsic motivation to improve IPE, especially since members rarely receive academic recognition from their own institutions for their SHAPED-related educational work. Furthermore, the association is spread across various regions without a clear base of operation, which makes recruitment of new members and long-term sustainability challenging. However, by maintaining the network through regular in-person meetings across Switzerland, SHAPED aims to counterbalance this. Currently celebrating its five year anniversary, it has so far stood the test of time.

## 5. Conclusion

Through an initiative of IPE enthusiastic students and young professionals, new intra- and extracurricular IP learning activities have been developed and implemented in Switzerland over the past five years. By being participatory, enjoyable, and realistic, these activities showed to positively influence students’ perception of IPE and achieved a high level of participant satisfaction. SHAPED is an example of how student leadership in IPE can overcome barriers and increase IPE. Letting students and young professionals develop their own IP teaching and learning activities can not only yield novel ideas but also enables cross-institutional collaboration with less administrative effort. However, for such initiatives to succeed and extend their reach to a broader student population, eventual intracurricular implementation should be pursued. Thus, the support of faculty (e.g. by recognizing their potential, allocating financial compensation and rewarding participation with academic credits) is paramount.

## Notes

### Funding

The goal of SHAPED has always been to provide its services free-of-charge for healthcare students. The needed funding is based on three pillars: membership-fees, sponsoring, and teaching-fees for intracurricular workshops at educational institutions. 

### Author’s ORCID

Fanny Mulder: [0009-0004-3382-242X]

## Acknowledgements

The authors would like to thank every current and past member of SHAPED for their time and effort put into this project, especially (but not exclusively) Tatjana Betschart, Aljoscha Noël Goetschi, Astrid Julen, Alessia Romer and Leanna Schoch.

Additionally, the authors would like to thank all of SHAPED’s sponsors and partner institutions for their continuous support.

## Competing interests

The authors declare that they have no competing interests. 

## Figures and Tables

**Table 1:**
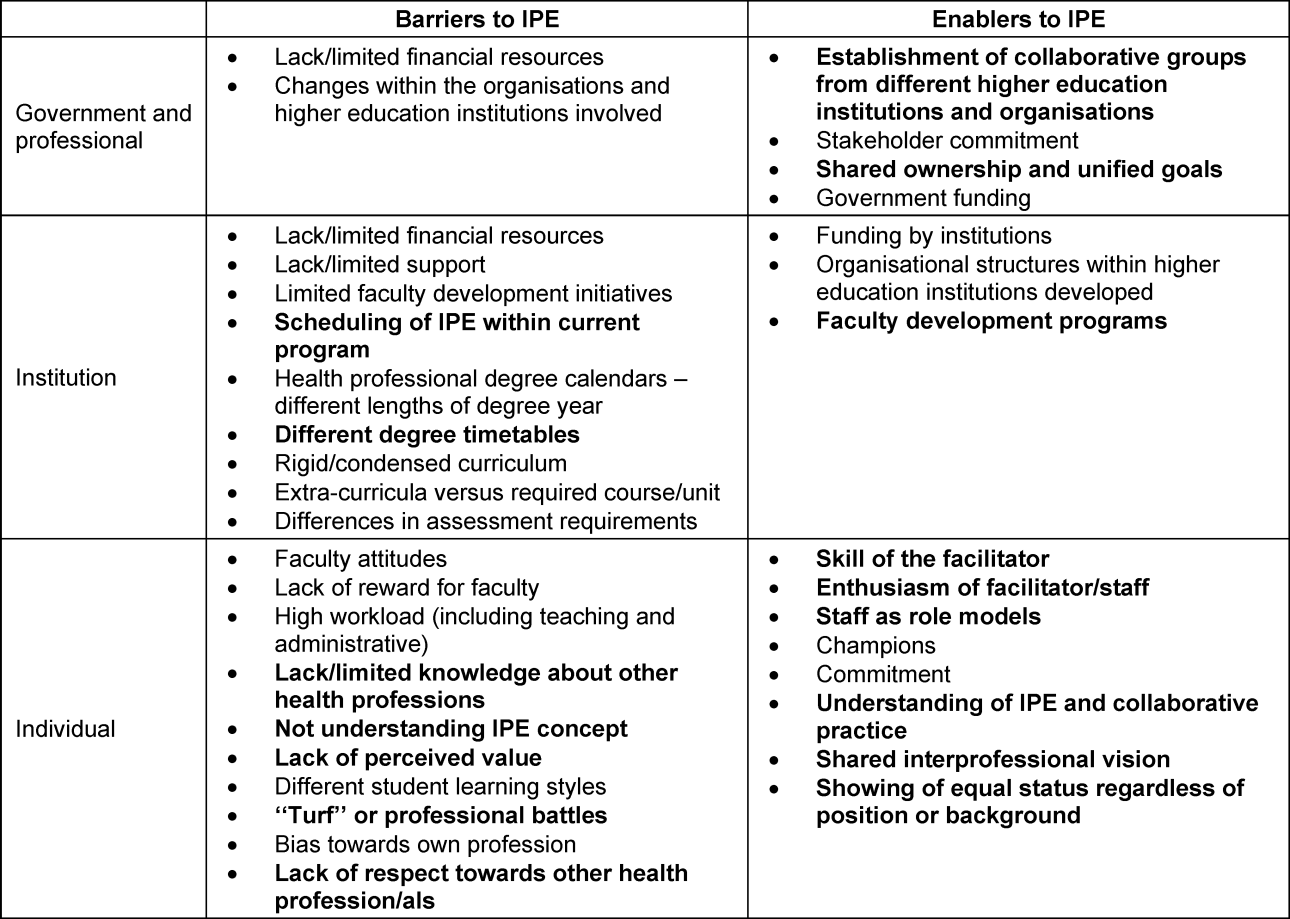
Barriers and enablers to interprofessional education (IPE) in higher education institutions according to Lawlis et al. [11] Barriers and Enablers to IPE in higher education institutions are drawn from Lawlis et al. (2014). Please refer to Tab. 2 and 3 of their article for further reference. Marked in bold are barriers overcome and enablers fostered by SHAPED as an interprofessional students’ and young professionals’ association.

**Table 2:**
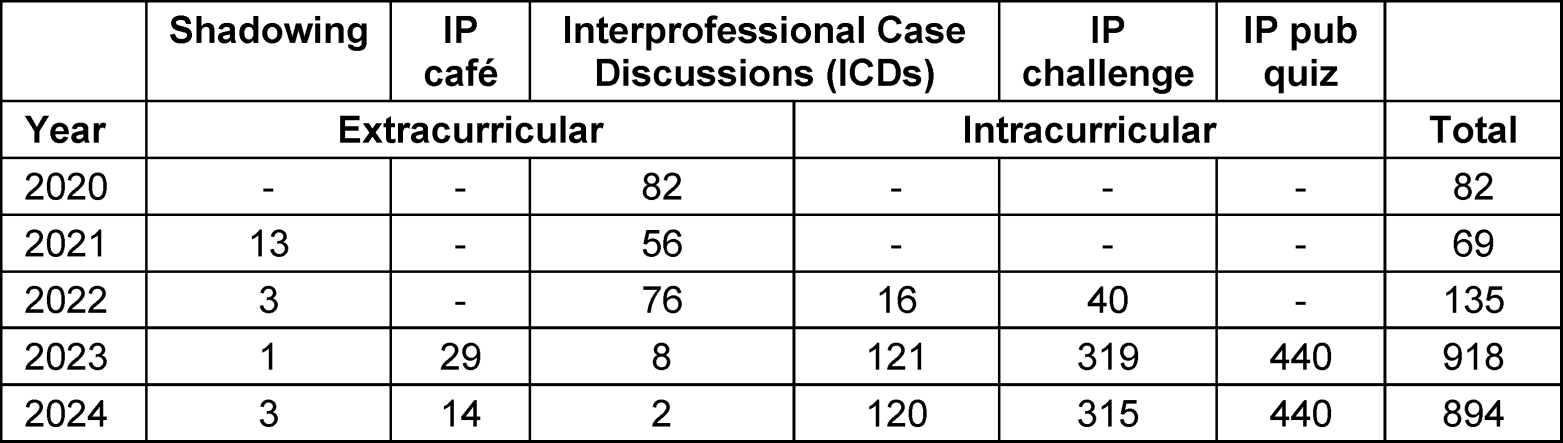
Participation in SHAPED’s IPE activities from 2020-2024

**Table 3:**
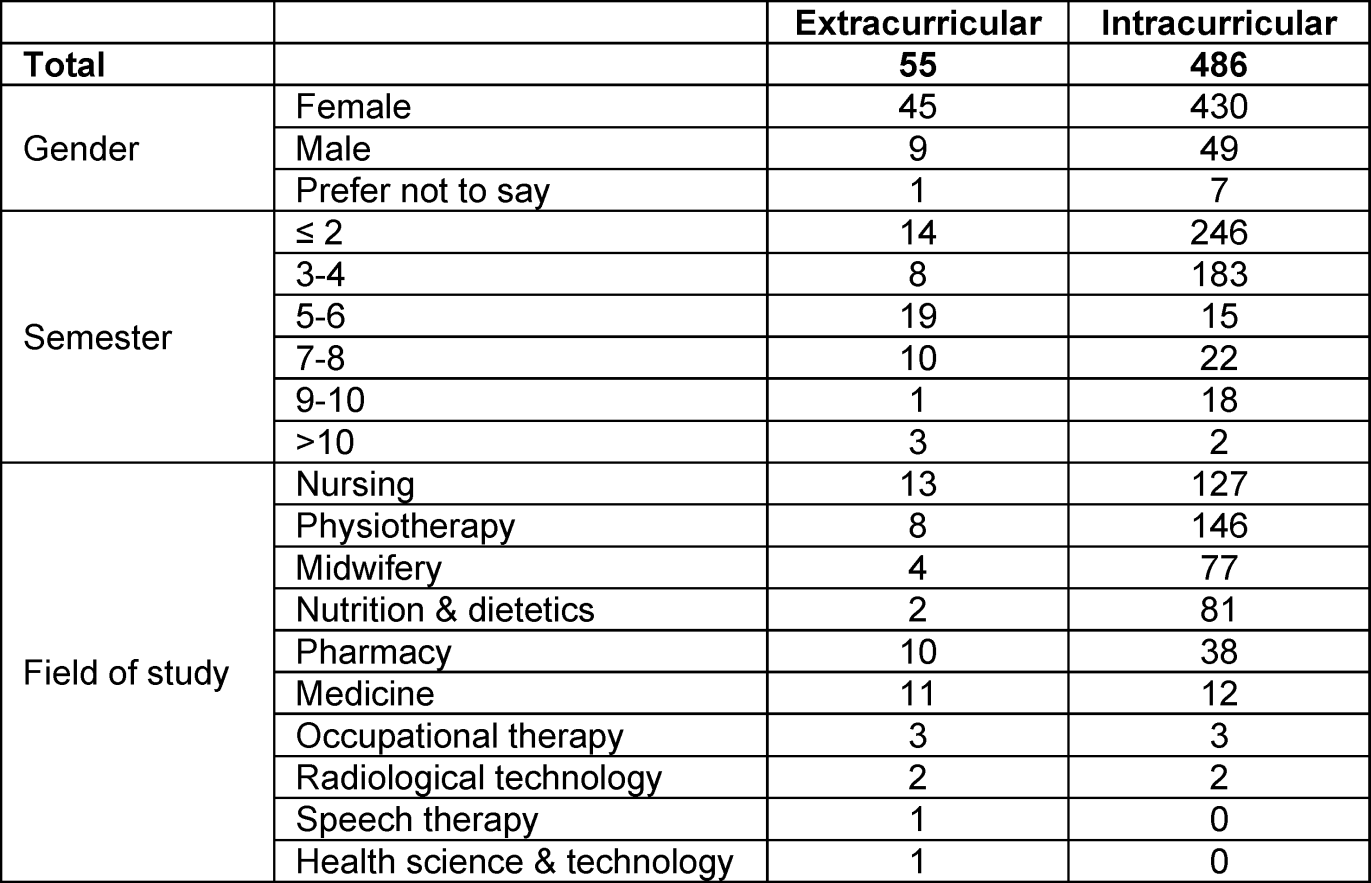
Characteristics of participants that completed the SPICE-R pre- and post-intervention
